# Supplementary material for: Patterns of Intron Gain and Loss in Fungi
Source: PLoS Biol. 2004 Nov 30;2(12):e422. doi: 10.1371/journal.pbio.0020422 (PMC532390; doi:10.1371/journal.pbio.0020422)
Supplement: Table S1 — Also available at http://genes.mit.edu/NielsenEtAl/. (4.3 MB ZIP). [file pbio.0020422.st001.zip › NielsenEtAl/html/876.html]

AN4965.1.NCU04766.1.MG02476.1.FG08474.1


```
 CLUSTAL W (1.82) Multiple Sequence Alignments - Introns Inserted


Sequence 1: NCU04766.1	2098 aa
Sequence 2: FG08474.1	2188 aa
Sequence 3: MG02476.1	2213 aa
Sequence 4: AN4965.1	2317 aa
Alignment Length: 2377 aa
Number Identitical Residues: 622 aa
Alignment Score (without introns) 39152


MG02476.1 	------------------------------------------------------------
NCU04766.1	------------------------------------------------------------
FG08474.1 	------------------------------------------------------------
AN4965.1  	MTFPPPPPSSQSSIASGTDKSSSTRPTRSQQSPWGPPVSQSSVRRGLTPLATNLSNPSVS
          	 : .....::.:: ::.:..:::: .: :..:. ... :.::   . :. ::. :..: :

MG02476.1 	-------------MQGITYSVVSWLAYLSEATKYE0ILMGPPPPRTG-------TFSPNN
NCU04766.1	-----------------------------------~------------------------
FG08474.1 	-----------------------------------~---MVPPPRAG-------SFSPNP
AN4965.1  	GRGQPQSNSPGPGVSTSSPLTSSFSAVLSSSRGHP~LGRGVPSPASTPGPFASYQSGSQQ
          	. ....:.:.... .  :    :  :  :.:          ... : ... ::   ... 

MG02476.1 	PQALQTGSSHSP-----------HPSLTGGLSAG-----------------------ASP
NCU04766.1	------------------------------------------------------------
FG08474.1 	AQTLQTGASHSP-----------HPSQAAILSAG-----------------------ASP
AN4965.1  	PQSLPSQSLSSPKIRAHTPSSGSHLASAAGSVAGGGGSGGGGGGTGSSRGATFSPLLSGT
          	..:  : :  :..  : :.::.:  : :.   :....:......:.:: .:: :.  :..

MG02476.1 	STSSPTGT---------------SVSKISIAQVYLLLSTIKEDKDDPTKWDLHTEQLRK0
NCU04766.1	-----------------------------------------------------------~
FG08474.1 	SASSPTGTN--------------SLTKIVVAQVYLLLSTIKEDKDR-AKWELQVDQLKK0
AN4965.1  	TVNSPTGFPPDKPGSVAAHGSQSSLAKISIAQVFLLLDSITE-KEGREKWETKAAQIHK0
          	: .:.:.  .....: :: .:.:: :.   :.     .: .. ..   . .    .  . 

MG02476.1 	LIDDNGMEVFTRYFARLVAGNASQIFPGISRPGSNAGNIHILAGEMRKIAHEPEQARKIA
NCU04766.1	------MEGFRSYFQRLVVGNAGQIFPNQKWQGVNSTTYHQLVAEVKKLSQDFEQSRRIA
FG08474.1 	LIDEHGMEVFSKYFTRLVAGNAPQIFPGLNRPVSNPGNYHILVGEMRKISHDVDQASKIA
AN4965.1  	LVESNGMEVFSKYFRRLLTGNAPQIFPGLNKSVENAGNYPLLVQEMQKVSQDIEQAQKIA
          	  .. .** *  ** **:.*** ****. .    *. .   *. *::*:::: :*: :**

MG02476.1 	ESIEQGTEDIFRDFDLSTFMEHFKLDPLEKTILALAFKLGTRSDLRTKA1DAILSTNYPT
NCU04766.1	EAIETGTEDLFRDFDLSTFMEHFKLDAFEKTVLALAFKLGSKPDLKTKA1DAILSTNFPM
FG08474.1 	ESIESGTEDIFRDFDLSTFMEYFKLDALEKTLLALAFKLGSRADLKTKA1DAILSTNFPS
AN4965.1  	ETVDASEGDIFRDFDLSTFLDHFKLDPIVKVSLALTFKTANKSDLRVKA1DAILANSITP
          	*::: .  *:*********:::****.: *. ***:** ..:.**:.** ****:.. . 

MG02476.1 	FLNILSR-PAGEHADLSPSFISLIIDRFIQEHPPTFATQAKSELILKVSARFAQ--ADQT
NCU04766.1	FGNILSK-SSGTHTMLGPDFIAGLLDRFIQVHPPNFNAAAKKELEHNVQSRYIQGNPDSN
FG08474.1 	FLDIISRPTLDAHADLSPPFIAMIVDRFLQYHPPSFNASARRDLETSVTKRWLGQTPEQT
AN4965.1  	FLQSLAS-PSDASKDFNNSFIGITIERFILYPPRNFTDDVKSKLLYAASLRYQALGVDMP
          	* : ::  . .    :.  **.  ::**:   * .*   .: .*   .  *:     :  

MG02476.1 	--PPPEVMAALDLLRILGDKSSNALTMYIKKTGAEFTRDPDVCVKYLQNRPSNAPLGEEL
NCU04766.1	PLPPSQVLSALDLMRLLADRPPNALTLYIHKTGQDFTENEETCYNYLRNRPANCQLSEEQ
FG08474.1 	--PPSEILAALDLSRVLADRPSNGLVKYIQKTGIELTRDEETCVSYLQNRPSSLQLGPEQ
AN4965.1  	----FEVASALQMFNFVN--PKYALVRQLHSRGPRATSSSD-AVNDTVSAAGPEFWSEEH
          	     :: :**:: ..:   .  .*.  ::. *   * . : . .   . ..    . * 

MG02476.1 	VANALMYTTISQAP-KHNPAVLVRALRQIVG-STFRWQAVVSCFDQCDSRVSSEQFLRLF
NCU04766.1	VSHALMYTTISQTP-PHNPSVLVTALRRVLP-GTFRWQDVVSYFDHSGARITAPQFLRLY
FG08474.1 	ISAAMTFTAISQTS-QQDPSILVGALRQVLP-KTFDWMEVIVLFDQPSARVSSQQFLRLY
AN4965.1  	IASALLFLVLSQYWREYSLETFLAAVRFHYGGKQINWSLIFRHFDREGLRLDARQFAKLY
          	:: *: : .:**     .   :: *:*    .  : *  :.  **: . *: : ** :*:

MG02476.1 	NAVVPVAQEDTS-FDIQQLWGGNWENTEAQLSFICALTSLGPEQLDITSVPGVQTTLTLD
NCU04766.1	NALLPIAQDDSQGFDIQRLWGGEWENPETQMSFVCAYTSLLPEQLDATTIPGLKPTFTLD
FG08474.1 	QAFLPIAQDSKSGFDIQRLWGGVWRNPEAQLSFVSAYASLVPEQLDATTIPGLQRSITID
AN4965.1  	AALSPIAAEDST-LDVQQLWGGEWEHRDTQMSFLTAFINS---RTDASQIPNLRATFPHD
          	 *. *:* :..  :*:*:**** *.: ::*:**: *  .    : * : :*.:: ::. *

MG02476.1 	DYSQSAPAVRERASVAVKHPLVSMAAILAIFHVALKSTPASDTPEAKRLFQQVVVPNLDI
NCU04766.1	DYAQAPQMIRETAAYAVRHPLVSEAAMSAIFNVALHSTHASKCTEAKRLFQDVVVPQVAI
FG08474.1 	DYANSPPNVQERAAVAVKHPLVSVAALSSIFNVALSSVMASTSIEAKRLFQEVVVPNLDI
AN4965.1  	FFSDGPEPVRLQGERAAKSPLRSLDATRAIFDLALYSQAAWAAAESQMLIKAVVQYDLPV
          	 :::..  ::  .  *.: ** *  *  :**.:** *  *    *:: *:: **  :: :

MG02476.1 	FVVSAFNVP-KPWPGVTDETLTTLFDNFLYER-SPWYDFVLDSLWRKDSAWVRQRLVDAH
NCU04766.1	FVVSAFRVP-KPWPKLAEDALVDLFIESLWAAKSPETDFVMNSLWRWDKDWVKMRLIDAH
FG08474.1 	FLVSAFEVPRQHWAPMAMETLGSLFENFLYKR-SPEYDFVLDSLWRKDKDWVIQRLIDAH
AN4965.1  	FLCSALALP-QPWTSVQQSFLLRTLAVFIMKQ-EEGYQLALHGAWRQDKQWVADQLFAMF
          	*: **: :* : *. :  . *   :   :    .   ::.:.. ** *. **  :*.  .

MG02476.1 	ALRPHELPYIVKHAVNHRWVDQLVSMTTGFGLDLAAMAHAEGRVDFAELARNSGER-QQD
NCU04766.1	AHRPICLPQIFEHSIRQGWLEELVYLINGFGFDLTALAHAEGYLDLQEWAQKNAER-STE
FG08474.1 	AIKPVDLPLVFEHAVKHNWLDSLVYLPNGFGIDLAALAHAEGYLDLSKWARNNAER-SAE
AN4965.1  	TQDPTSTAAIYEHAIEYNWLEYLLGYTNGLAMDLACYAHRKGPFELEQWVRNAASKGLMD
          	:  *   . : :*::.  *:: *:   .*:.:**:. ** :* .:: : .:: ..:.  :

MG02476.1 	LARYLLQFLAIKAEQELKFQR-QPDGNVPIRTTTPLRVKTVYVLLSMLSDFLPKHQPIPE
NCU04766.1	MASALLQFLMIKANLELQYQRSGPG---APKQSTTLQVRTVSAMLQILEEFLPK-SPIPE
FG08474.1 	IARTLVQFLMIKANLESQYQRGGADGQPAIKNSTTLQVRTVSALLRILEDFLPS-PPVPE
AN4965.1  	MGTLLSKYLRIKADDELRVQR----REQLAPQMVSLSVKTVFTLLSVLEEYVGD---PQN
          	:.  * ::* ***: * : **     .      ..* *:** .:* :*.::: .     :

MG02476.1 	LIRVQRTCITVYPRLINYGGDFDDIIDANGREGNALPPAANAKMELHYKKMYSDEVEVRE
NCU04766.1	LIVVQRQCITAYPRLINYGEGFDDIIDANGRDGNSLPQAANVRMEEHYKKMYSDEVAVRT
FG08474.1 	LIVVQRHCIIAYPRLINYGEGYDDLIDANGRDGNALSPIANGRMEEHYKKMYSEEVQVRN
AN4965.1  	LTPVQRICIQTYPRLINYGEGFDDIIDANGESGNALPEPVDKKMQELFGKMYHEELSLRE
          	*  *** ** .******** .:**:*****..**:*.  .: :*:  : *** :*: :* 

MG02476.1 	IVQVLENYKRSRDTLDQDIFACMIHGLFDEYIHFAGYPLEALATTAVLFGGIIAQKLIAD
NCU04766.1	IVEVLDQYKHSRDPLEQDTFACMIHGLFDEYAHYENYPLEALATTAVLFGGIISHKLVSE
FG08474.1 	IVEVLSQYKHSRDPLDQDVFSCMIHGLFDEYSHYGDYPLEALATTAVLFGGIISHKLVSN
AN4965.1  	ILELMRQYKSSRDPAEQDLFACMVHGLIDEYHCYHEYPLEALTKTAVMFGGIINFRLVDG
          	*:::: :** ***. :** *:**:***:***  :  ******:.***:*****  :*:  

MG02476.1 	IPLEVGLGMILQAVQENTPNEAMYKFGLQALMQLFGRLKEWPGFCAQLLQVPGLQGTEAW
NCU04766.1	LPLKIGLGMILEAVRDHQPQEPMYKFGLQALLQLLPRLREWPGFCTQLLLIPGLQNTEAR
FG08474.1 	LPLQIGLGMILEAVRDHSQEDPMYKFGLQALMQLFVRFREWPSFCRQLCQIPGLHNTEAF
AN4965.1  	ITLKVGLGMILEAVREHDIHDPMYKFGVEAIEQLINRLPEWVGFCHLLLQIPSLQGSPIF
          	:.*::******:**:::  .:.*****::*: **: *: ** .**  *  :*.*:.:   

MG02476.1 	KKAEDVIRNQEESMASTRNGG----------DALANGNAEEGGENEHHPPPFTAINVDRR
NCU04766.1	KKAEDVLREHEEETMRARGGA-LPLGATMGNDNMTNGNLDEAHGSEQHPPPFASVNVDPP
FG08474.1 	RKAEEVVREHEEELSRGRNGAGTPHGLGFQGETFPNGNVEELASADRQAPAFASLNVDPP
AN4965.1  	QKAEEVLREQGSQVRDENGRQ-----DNTTGGSITNGNVDESAAPDGANRKFVSIHVDPP
          	:***:*:*:: ..    ..           .  :.*** :*    :     *.:::**  

MG02476.1 	PADPGFEDPDDDTQGKIQFVLNNITENTLQVMFKELAEMLERGHKQWFASHLVEERAKMQ
NCU04766.1	PPGVIYEDPTAEAQGKIQFFLNNLTETTLQTMYKDLRDMLETKHQQWFASHLVEERAKMQ
FG08474.1 	PPDVEFEDPDEGAQDKVQFVLNNITEGTIREMCQELRETMERRHQQWFASHLVEERAKMQ
AN4965.1  	LRPEIYQDPDEEVQDKILFVLNNVSEQNIDEKLRDLTDVLKDPHHQWFASYLVEERAKLQ
          	     ::**   .*.*: *.***::* .:    ::* : ::  *:*****:*******:*

MG02476.1 	PNYHDVYLKLVEQFGDKSLWAEVLRETYISVSRILNAEATAHNSTDRMHLKNLGGWLGLL
NCU04766.1	PNYHQVYLDLVKMFEDKSLWAEVLRETYVSVQRMLNSDVTLQNSTERTHLKNLGGWLGLL
FG08474.1 	PNYHHVYLELVRLLEDRALWSEVLRETYVSVCRMLNSEATMQNSTERTHLKNLGGWLGLL
AN4965.1  	PNFQQLYLDLLDRIDDKILWVEVLRETYASTSKLLNSEATLGSSTERGHLKNLGSWLGSL
          	**::.:**.*:  : *: ** ******* *. ::**::.*  .**:* ******.*** *

MG02476.1 	TLARDKPIKHKNIAFKQLLMEAHDTKRLLVVIPFVCKVLIQGKKSTVFQKQNPWLMDIIH
NCU04766.1	TLARNKPIKHRNIAFKQLLMEAHDTKRLLIVIPFVCKVLAQGASSEVFQPPNPWLMDIIH
FG08474.1 	TLARDRPIKHKNIAFKQLLIEAHDTKRLIVVIPFVCKVLTQGATSAVFRPPNPWLMDIIH
AN4965.1  	TIAKDKPIKHKHIWFKGLLLEAYDSQRLTIVIPFTCKVLVQATKSTVFKPPNPWLMDIIA
          	*:*:::****::* ** **:**:*::** :****.**** *. .* **:  ******** 

MG02476.1 	LLIELYHNAELKLNLKFEIEVLCKGLELDHKSIEPSGELLNR-VMTEDAV-DLLPADTLE
NCU04766.1	LLIELYHNAELKLNLKFEIEVLCKGLKLDHKSIEPSGEILNR-VPVEEVP-DLN-GQELE
FG08474.1 	LLIELYHNAELKLNLKFEIEVLCKGLNLDHKSIEPSGEILNRPVVVEEPA-DVLPTEQLD
AN4965.1  	LLMELYHFAELKLNLKFEIEVLCKDLEIDHKAIEPSVIIRDRSTTIEESLSTANIPEGLE
          	**:**** ****************.*::***:****  : :*..  *:  :     : *:

MG02476.1 	PFENLSLNGLAPGVGSG-LSPHAVAPSIPDLTPFLQIPPVNEMVVSHERMTQITNQAITK
NCU04766.1	PYDNTSINGMGSVAGG---LPAHASLPIPDLSASLSIPPT--EVIDQAKLHSITSQAVTR
FG08474.1 	AFENLSLNGIGSTVGAG-LSPQTLAPTIPDLGPLINIPPTNEMVVSTTRLHEIVRTALSR
AN4965.1  	AFEDMALTSISQSIRNERLSPAAILSTLPSLDKILVLPPSASSMVDSNVLRQIVHSAVER
          	.::: ::..:.         *     .:*.*   : :**  . ::.   : .*.  *: :

MG02476.1 	ALSDIIGPVVDRSVTIAAISTQQMIHKDFGTEPDENRVRTSAINMVKATAGSLALVTSRE
NCU04766.1	ALQEIIQPVVDRSVTIAAIATQQMIHKDFATEPDENRVRDAAINMVKSTAGSLALVTSKE
FG08474.1 	ALQDIIQPVVDRSVTIAAISTQQMIHKDFAIEPDENRVRTSAISMVKATAGSLALVTSKE
AN4965.1  	AIAEIITPVVERSVTIASISTVQLVSKDFAMELDEEKFRHAAANMVRQLAGSLALVTCKE
          	*: :** ***:******:*:* *:: ***. * **::.* :* .**:  ********.:*

MG02476.1 	PLRVNIGNYMRSLSSDLP-QGLPEGNIHMAVNMNLDLASREIEKAAEERAVPEIEEMLEL
NCU04766.1	PLRSNMANYLRALSAEIG-LVLPEGIIMLCINSNLDLASSVIEKSAEDRAVPEIEEMIQP
FG08474.1 	PLRANFTNYMRNLSNDLP-QGLPEGTIIMCVNSNLDLACNIIEKQAEERAVPEIEEMIES
AN4965.1  	PLKVSMTNYIRMIQQEYSDQPMPEGLILMCVNDNLDAACGIVEKAAEEKSLPEIEKVIES
          	**: .: **:* :. :  .  :*** * :.:* *** *.  :** **::::****:::: 

MG02476.1 	ELEARRRHRATRPNEPYVDAGLSRWAWTIPNPYKLSPNMSGLNPEQMAIYENFARQQPRT
NCU04766.1	EIEARRRHRAQRPNEPYMDPGLTRWAWTIPHPFKLNPSMTGLNAEQMAIYDDFARQ-PRA
FG08474.1 	ELEARRRHRIQRPTDPYFDSSLSRWAMAIPHPYKLSPNINGLNHEQMAIYEDFARQ-PRS
AN4965.1  	QLEARRRHRAARPSEPFIDPSMTRWGLFIPEPYRQTP--GGLNKEQLAIYEEFARQ----
          	::*******  **.:*:.*..::**.  **.*:: .*   *** **:***::****    

MG02476.1 	TAASAPPTHAPSQSDASRSLANEVLQDQYNSVPTLPTPAETPAIPPMGSQQIGPYSS-QQ
NCU04766.1	VASTTGPSHAASTSDAR-SLANEVLGDQFNTISALPTPAETPSLP-HLGGQLQHYP---Q
FG08474.1 	ATLPT-PSHGQSASDATRSMANEVLQEPFATVPNIPTPAETPSIQQHLGAQMQPYAPVHS
AN4965.1  	-ARGPGTTHAQNVSTDSGRQLPDVLQEPYPPIPNLSTPAEQPAVP-HRTPQPQQDTRLQQ
          	 :  . .:*. . *        :** : : .:. :.**** *::      *    .   .

MG02476.1 	HSSSMVNGRQHGL-NLDARALAERVQKFLLELQRVAMDAEEEHFSELPRPHQILDIVDAL
NCU04766.1	GSGGLANGRQAGLNQVDARTIADRVNKLLESLTAAATGATEEHFNELPRAHQVLDIIDAL
FG08474.1 	GANTMSNGRSPGF-QMNVQSLAERVNKLLQELVRVASESPEEHFQGLPRPHSLLDVVDAL
AN4965.1  	SALVGTSSQLNGF--LETQNPRDRVESLVSDLQQAARNATEEHVKDLGRDSAVLQEYNQA
          	 :    ..:  *:  ::.:   :**:.:: .*  .*  : ***.. * *   :*:  :  

MG02476.1 	VQLLIKAQQASEDLSVYAAELISQSLFGNPAEKSLVTETFVHVLNTLRKIASPALNLRVT
NCU04766.1	VQLIIKTQQTSEEFAAYAATQIAQLIFRQPGDT-LLLESLVHVLETLRKIAGPATSQQIR
FG08474.1 	VQHIIKTSQNSEEFAIYAAEQITGLIFSQVDDN-LTLESLVHVLETLRKISGPALNSRIR
AN4965.1  	LRSIVASP-NGEEVARLTALKACTSLYSQTKST-LEIEVLVHLLAKLCDMS-ALVARYTW
          	:: :: :   .*:.:  :*      :: :  .. *  * :**:* .* .:: .       

MG02476.1 	NFFQQQTGEHLRNLTLISSLVNTDLLNWRHVDNAIATAIKERKSGSIEFLEKVMDLTVFN
NCU04766.1	SLFHQQPAEYFLNLSLIAALLGTDLLDWRSIDAAMSMALQQRKEGSVEFLEQLVDLTLLS
FG08474.1 	TLFSQQAGSTFLNLPLLAALVRTDLLEWRSIDLAMTKALQARKEGSLDFLEHMLDLTLLD
AN4965.1  	TVLAEVDDEHMFNVPVTVALIDAGLLDIRRVDMILSRLILQKNAAALDVLAELMDQILFN
          	..: :   . : *:.:  :*: :.**: * :*  ::  :  :: .:::.* .::*  ::.

MG02476.1 	DTPVLLFVEFVHSLDEAWTWIQEDSELAEGKSLQAKVLAPANEPRAGLSDDELASLRQDQ
NCU04766.1	ESPLALYADFVRSLEEAWAWISENPDVPGGQSLKSKILAPPPDLPEGLTPAEILAIRQDQ
FG08474.1 	ARPIALYADFASTLEAAWAWVLEDPDSSAGQRIKSKLVN--SGLTRPTPVDNQALFKHDQ
AN4965.1  	EEPSALRSDFSGSLEAMSKWLAEDSGLARAKEIIKKLRDSGIPEVVNPLLSDKARSKRDQ
          	  *  *  :*  :*:    *: *:.  . .: :  *:  .           :    ::**

MG02476.1 	MEYVFEEWVRLFTNPWAS-EKFGFIFVQQMYGRGVISSKDDIATFTRIAMDKSVDICELN
NCU04766.1	MEYVFDEWVHLCNNPAASSSKSSTIFVQQMRSRGVIASTDDFLMFARLAIDKSVDRFEHV
FG08474.1 	IEYVFDEWIRLCHNHNAS-DKIIAAFVQQLQNNQVLQTRDDFFVFIRVALDISADRFEYI
AN4965.1  	MEYIFTEWIGIYKAPGAV-DRTYYAFLKDMHQRQVMANQEDSALFFRLSIDISVARFEHE
          	:**:* **: :     *  .:    *::::  . *: . :*   * *:::* *.   *  

MG02476.1 	AHAG-GSVSDGYLGVDSLSRLVSTLMKLPVENPSSGQVSRLSVLECALSVSVLLANHHQM
NCU04766.1	VHLN-GTITEAYQAIDALGKMILIFFQSHEDNADG-QTARAAFIDSILALGVLVLNSHHV
FG08474.1 	VRT--GSLGDAYVMVDALSKLIGTFIG---MGFEP-TSTRPAFIDSVLSLVELILTNHHV
AN4965.1  	SQNPSGSLDEAFLYIDALAKLVVLLVKFQGEGTGPVKTSKSAYFNSILSVLLLVLNHHQV
          	 :  :*:: :.:  :*:*.:::  :.     .      :: : ::. *::  *: . *::

MG02476.1 	TRGELFNQRVFFRFFSVLLHEIGAMENLLSEVERHDITLLFARRLDDLGPSVMPGFVYAW
NCU04766.1	KRGDAFNSRVFVRFFSYLLHGIHSL-EELPENERDRFMLSFAERLYDLRPSLYPGFVLGW
FG08474.1 	KRGEELNQRVFFRLLSMLFHEIQTISEALPEDERREVLVKMAVRLNKIGPAFAPGFIYGW
AN4965.1  	MRGEAFNQRVFFRLFSSILCEYSMS-GLQHSDQHQEMMFTLANKLLSLQPKYYPSFVYGW
          	 **: :*.***.*::* ::           . ::  . . :* :* .: *   *.*: .*

MG02476.1 	VSLLQHKEFLPFIFKVPGNAGWPLFTRLLTQLLSFSGEQLKALNVSNLAKDLYRGTLKFM
NCU04766.1	LTLVTHRVFQPAMLQMANNAGWEAYTKILRDLLSYQGELLKPLELSIFAKEMYQAVLKLL
FG08474.1 	LSLVQHRAFMPSILQLPDGAGWTPFATLVCQLLDTLGDQLKVFEVSNIAKDIYRATFKLL
AN4965.1  	FSLVSHRFFMSSMLNMPDRAGWGPYCEIMQVQLSYIGEQLKSTTMTLVTMDLYRGMLRIL
          	.:*: *: * . ::::.. ***  :  ::   *.  *: **   :: .: ::*:. ::::

MG02476.1 	LVLQHDFPDYLAANYIEICRSIPAHCTQLTNMVLVATPNTHSKMPDPLHSGVQVDRIDES
NCU04766.1	IILGHDFPDYVSANHVLLCQSLPPHATQLRNMILLATPASAAKPTDAFQPGLKLDQIADI
FG08474.1 	IILHHDFPEFVAANHIRLCASIPPHCTQLINAVLAATPHQIPRINDSNVK----DQADEI
AN4965.1  	LILHHDFPEFVAENHFQFCNVIPAHCAQLRNLVLSAYPSSFQKLPDPFREGLKVERLEEM
          	::* ****:::: *:. :*  :*.*.:** * :* * *    :  *.   . . ::  : 

MG02476.1 	REPPFTMEDPTEFLRQTGLQGVLDQCLQSG-PSEDAIAHITHAINRPETRYTIYGNVPLS
NCU04766.1	KEAAISVYDPAEQLRQLGLLDILDQALQNG-PSEDAIAQITHAITRTGSGTTDYGFVPID
FG08474.1 	HVHPGLLHEATSVLQDRGLLNAIDQALQSG-PTEDIVANITHAMAQDTRKTTTYGHVPVD
AN4965.1  	REAPRIAGDTAGPLLRAGIKNVIDNALQSNNVSDSVIQQIRDAVYDTESKESGLFHAPIN
          	:  .    :.:  *   *: . :*:.**... ::. : :* .*:       :    .*:.

MG02476.1 	VNLAVIEAVVAYLGLHAVVRVQKGGQP--FMSKAFDISTLSILVHELSPEGRYFVLSSVV
NCU04766.1	VNRQVIDAVITHIGNFAVNRAATRNDASVFVPGAPDLKTLQMLITELSPDTRYFVLNSMA
FG08474.1 	ANMAIIDAVNIYTGQHAAEKAAQAGTTVSVTGDEHEVTLLSMILHELSAEARYYLLVSVV
AN4965.1  	INLLLVNALALYVGQGAVSVNESKGNTRAAFESSAHSALLEKLAKILRPEARYYLLSAMA
          	 *  :::*:  : *  *.      . .        .   *. :   * .: **::* ::.

MG02476.1 	HRLRYANAHTTFFSQALLEIFGTDMA--DAEETEIRQQICRILLERLVGFFPQPWGLLVT
NCU04766.1	NWLRFTSAHTHYFIQVFLDFFGHDVASTDPEEMDIRQQIVRILLERLIGYWPYPWGLIIT
FG08474.1 	NQLRFPNAHTTFFRQALLHFFGKDFD--DPEENEIRQEITRVLLERLVSFWPQPWGLLYT
AN4965.1  	NQLRYPNSHTYFFSFAILRLFGTDYT--EQDESDIRQQIIRVLLERLIVHRPHPWGLIIT
          	: **:..:** :*  .:* :** *    : :* :***:* *:*****: . * ****: *

MG02476.1 	VAELIKNDKYQFFELPFIKSSPD0LVAASDGHVCKRSTQSV
NCU04766.1	TMELVKNEKYNFFELPFIKATPE~VRRVFEDKVRQH-----
FG08474.1 	VVELMKNEKYMFFDLPFIKATPD0VAEKFSRVLTQA-----
AN4965.1  	LQELLQNSSYAFFHLPFIQAAPE0L----------------
          	  **::*..* **.****:::*: :
```
